# Development of a Machine Learning Model for Predicting Weaning Outcomes Based Solely on Continuous Ventilator Parameters during Spontaneous Breathing Trials

**DOI:** 10.3390/bioengineering10101163

**Published:** 2023-10-05

**Authors:** Ji Eun Park, Do Young Kim, Ji Won Park, Yun Jung Jung, Keu Sung Lee, Joo Hun Park, Seung Soo Sheen, Kwang Joo Park, Myung Hoon Sunwoo, Wou Young Chung

**Affiliations:** 1 Department of Pulmonary and Critical Care Medicine, Ajou University School of Medicine, Suwon 16499, Republic of Korea; petitprince012@ajou.ac.kr (J.E.P.);; 2 Land Combat System Center, Hanwha Systems, Sungnam 13524, Republic of Korea; kimdo0@hanwha.com; 3 Department of Electrical and Computer Engineering, Ajou University, Suwon 16499, Republic of Korea; sunwoo@ajou.ac.kr

**Keywords:** ventilator weaning, machine learning, extubation, prediction, visual explanation

## Abstract

Discontinuing mechanical ventilation remains challenging. We developed a machine learning model to predict weaning outcomes using only continuous monitoring parameters obtained from ventilators during spontaneous breathing trials (SBTs). Patients who received mechanical ventilation in the medical intensive care unit at a tertiary university hospital from 2019–2021 were included in this study. During the SBTs, three waveforms and 25 numerical data were collected as input variables. The proposed convolutional neural network (CNN)-based weaning prediction model extracts features from input data with diverse lengths. Among 138 enrolled patients, 35 (25.4%) experienced weaning failure. The dataset was randomly divided into training and test sets (8:2 ratio). The area under the receiver operating characteristic curve for weaning success by the prediction model was 0.912 (95% confidence interval [CI], 0.795–1.000), with an area under the precision-recall curve of 0.767 (95% CI, 0.434–0.983). Furthermore, we used gradient-weighted class activation mapping technology to provide visual explanations of the model’s prediction, highlighting influential features. This tool can assist medical staff by providing intuitive information regarding readiness for extubation without requiring any additional data collection other than SBT data. The proposed predictive model can assist clinicians in making ventilator weaning decisions in real time, thereby improving patient outcomes.

## 1. Introduction

Estimating an appropriate weaning time from mechanical ventilation is an essential clinical decision in critical care. Premature attempts to extubate patients increase the risk of ventilator-associated pneumonia [1,2], prolonged intensive care unit (ICU) stay [3,4], and mortality [1,3,4]. Meanwhile, an unnecessarily prolonged duration of mechanical ventilation causes an enormous economic health burden [5,6] and is associated with deteriorated clinical outcomes [7,8]. Therefore, an accurate prediction tool for deciding when patients are ready for extubation is critical for managing patients with respiratory failure.

Previously proposed weaning indices have shown conflicting results, as over one-quarter of patients require reintubation despite meeting the criteria for such indices, such as the rapid shallow breathing index (RSBI) [9,10,11,12]. With the recent emergence and advancement of artificial intelligence, several studies have incorporated machine learning methods to facilitate efficient clinical judgments in the ICU, including those related to airway management [13], acute kidney injury [14,15], pressure ulcers [16], and mortality [17]. Pioneering studies have been conducted using machine learning methods that incorporate diverse features to predict the success of ventilator weaning [18,19,20,21]. These features include demographic information (i.e., age, sex), comorbidities, laboratory results (i.e., electrolyte levels, arterial blood gas analysis), vital signs (i.e., blood pressure, heart rate, respiratory rate), respiratory parameters (i.e., tidal volume, ventilator mode), medications (i.e., vasopressors, antibiotics), treatment type (transfusion, continuous renal replacement therapy), and clinical scores (i.e., Acute Physiologic and Chronic Health Evaluation II [APACHE II], RSBI). However, the predictive machine-learning models in use have limitations, such as the burden on medical staff to promptly collect bedside information, and the intricacies of processing this data contributes to the complexity.

In this study, we aimed to develop a predictive machine learning model for weaning outcomes which would directly analyze continuous ventilator data, such as raw waveforms and numerical monitoring parameters, during routinely performed spontaneous breathing trials (SBTs) before extubation. Furthermore, we attempted to visually depict the features that affect the predictive model’s results in the continuous waveform data. Unlike previous studies, this method eliminates the need for medical staff to collect or compute any additional information beyond that collected during SBTs. Furthermore, this tool instantly provides intuitive information regarding a patient’s readiness for extubation. To improve the efficiency and effectiveness of the weaning process, we focused only on the ventilator parameters collected during SBTs.

## 2. Materials and Methods

### 2.1. Data Sources and Participants

We enrolled patients sequentially admitted to the medical ICU at a Ajou University Hospital in South Korea between January 2019 and September 2021. Patients’ clinical data were obtained from electronic medical records to identify their baseline characteristics, and ventilator parameters were extracted by directly connecting the acquisition software (Hamilton Medical ventilator data logger, version 5.0, Bonaduz, Switzerland) to the ventilator during SBTs. Twenty-five types of numerical data and three types of waveform data were extracted as the ventilator parameters (Supplementary Table S1). Waveform data were sampled every 15 ms, and numerical data were collected during each breath. The predictive model for weaning outcomes relied solely on data from the ventilator waveform and numerical recordings during the SBT, without incorporating data from other sources.

This retrospective study used anonymized data and was approved by the Institutional Review Board of Ajou University Hospital (IRB No. AJOUIRB-MDB-2022-094), which waived the requirement for informed consent. This study was conducted in accordance with the Transparent Reporting of a multivariable prediction model for Individual Prognosis Or Diagnosis (TRIPOD) guidelines for prediction model development and validation [22].

Eligible participants included individuals aged 18 years or older who had undergone mechanical ventilation for more than 24 h and who met the weaning criteria. Weaning criteria included resolution or improvement of the underlying condition leading to intubation, hemodynamic stability (systolic blood pressure between 90 and 160 mmHg and heart rate below 140 beats per minute with low/no doses of vasopressors), stable neurological status (no deterioration in Glasgow Coma Scale (GCS) score within the last 24 h), respiratory stability (oxygen saturation above 90% with fraction of inspired oxygen [FiO_2_] not exceeding 0.4, respiratory rate below 35 breaths per minute, and spontaneous tidal volume above 5 mL per kg), and intact cough and gag reflexes [23,24,25,26]. Exclusion criteria included patients with tracheostomy, with a do-not-reintubate order, and without recorded ventilator data.

### 2.2. Study Design

Each patient received a 30 min SBT with a maximum pressure support ventilation of 6 cm H_2_O and positive end-expiratory pressure. The FiO_2_ was maintained at the same level as that before the SBT. Following the stable completion of the 30 min SBT, patients were extubated and provided with either a high-flow nasal cannula or an air entrainment mask for oxygen therapy. Patients unable to tolerate a SBT were maintained on mechanical ventilation. Failure to meet the SBT criteria included agitation, anxiety, deterioration of consciousness, a respiratory rate exceeding 35 breaths per minute or the use of accessory muscles, oxygen saturation levels below 90% (measured by pulse oximetry) with FiO_2_ above 0.5, heart rate surpassing 140 beats per minute or a 20% increase from baseline, systolic blood pressure below 90 mmHg, or the development of an arrhythmia.

Patients who underwent extubation were classified into two groups, the weaning success and weaning failure groups, based on their condition within 48 h after extubation. A patient was considered to be in the weaning success group if they maintained a stable condition for >48 h after extubation. In contrast, patients who required reintubation due to respiratory failure within 48 h after extubation were included in the weaning failure group. The criteria for respiratory failure were as follows: respiratory acidosis with a pH level of less than 7.3, partial pressure of carbon dioxide (PaCO_2_) levels higher than 45 mmHg, oxygen saturation levels below 90% with FiO_2_ levels above 0.5, respiratory rate exceeding 35 breaths per minute, deterioration of consciousness, severe agitation, or clinical signs of respiratory fatigue. The clinical data of all enrolled patients were reviewed by two critical care specialists (W.Y.C. and J.E.P.), who verified their inclusion in either the success or failure groups.

### 2.3. Proposed Weaning Prediction Model

Our proposed weaning prediction model was designed using two convolutional neural networks (CNNs) to extract features from multimodal input data with various sequence lengths. We adopted MobileNetV3-0.75 as the backbone [27], which was designed for mobile device environments, making our model adaptable to various hardware environments.

#### 2.3.1. Data Flow

**Figure 1:**
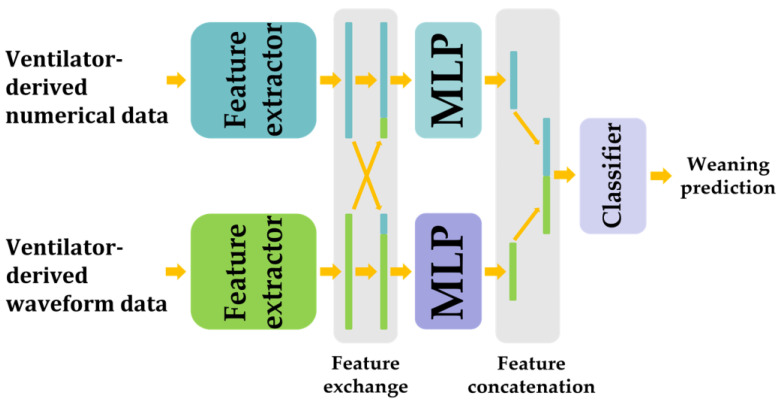
The overall architecture of the proposed network. Our model for predicting weaning uses two convolutional neural networks to analyze various data types with varying sequence lengths and extract important features. MLP, multi-layer perceptron.

The proposed model was applied by feeding the ventilator waveform and numerical data into the feature extractor. Subsequently, the feature extractor generated outputs, which were fused with other outputs from the feature extractors to create 20 of the 720 features. This fusion allows the proposed model to analyze the correlation between the ventilator numerical data and waveform data. The fused outputs were then fed into a multi-layer perceptron (MLP), in which the outputs were concatenated and fed into the classifier. Finally, through the classifier’s output, the proposed model provided a prediction for weaning success or failure from mechanical ventilation (Figure 1).

#### 2.3.2. Feature Extractor and Classifier

The MobileNetV3-Large 0.75 constitutes the feature extractor and classifier [27]. Table 1 lists the detailed operators [27]. The feature extractor consisted of two convolution layers, 15 bottlenecks, and a pooling layer. We then constructed the classifier using the remaining modules [27]. The output channel size of the last layer in the classifier was set at 1 for the binary classification task. The order of operations in the feature extractor and classifier are listed in Table 1.

#### 2.3.3. MLP and Subblock

**Figure 2:**
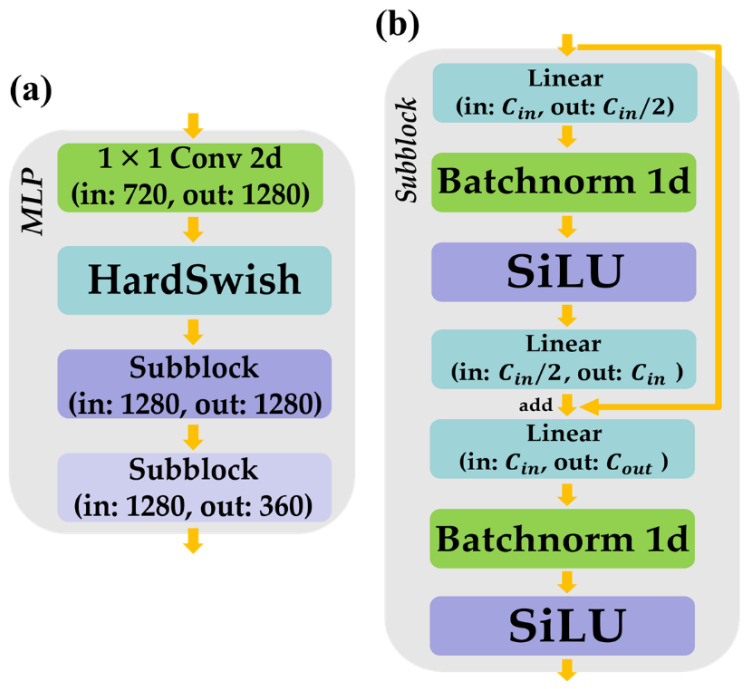
Proposed MLP and subblock, where “in” denotes “input channel size” and “out” denotes “output channel size.” (**a**) Structure of the MLP; (**b**) Structure of the subblock. Batchnorm, batch normalization; Conv, convolution; MLP, multi-layer perceptron; SiLU, sigmoid linear unit.

Figure 2a shows the proposed MLP architecture. The MLP encodes the fused feature shown in Figure 1 to a lower dimension. This module consists of four layers: 1×1 convolution, hard swish [27] as an activation function, and two sub-blocks. The order and input and output channel sizes of the layers are shown in Figure 2a.

Figure 2b shows the proposed subblock of the MLP. We proposed a sub-block inspired from previous studies [28,29]. The subblock uses a linear layer instead of a convolution layer to extract information from the one-dimensional input features. This module consists of linear layers, batch normalization [30], and two sigmoid linear units [31]. The order of these layers is illustrated in Figure 2b. The subblock adds its input feature and the output of the second linear layer using a skip connection [28]. This summation prevents information loss in the forward path and gradient loss in the backward path [28].

### 2.4. Training and Validation

The dataset was divided randomly in an 8:2 ratio into training and test sets to develop a predictive model. The training set was used to optimize the model parameters and select the best model hyperparameters. The model performance was evaluated using an independent holdout test set. This approach allowed us to evaluate the performance of our model effectively and optimize its parameters. In the holdout test dataset, the performance of the predictive model was assessed using nine metrics: area under the receiver operating characteristic curve (AUROC), area under the precision-recall curve (AUPRC), sensitivity, specificity, positive predictive value, negative predictive value, accuracy, F1 score, and parameter count. Furthermore, the RSBI, which is already used in clinical practice, was used to accurately compare and evaluate the model’s performance.

The waveform and numerical data from the ventilator contained 3 and 25 features, respectively. Each feature of the data was normalized with a time axis using min-max scaling. Although our model was designed to process various sequence lengths of input data, one such batch could not be used for model training. In this study, the input sequence length for training was set to 13,000 and 3500 steps for ventilator waveform and numerical data, respectively. After normalization, the input training data were randomly cropped to a defined size before being fed into the proposed model. Finally, the proposed model was designed to process the input data independent of the sequence length using a CNN. Entire sequences of ventilator numerical and waveform data were used as model inputs.

The proposed model used a binary cross-entropy function with a sigmoid function as the loss function. The weights of the model were updated using the AdamW [32] optimizer equation as follows: $learning\;rate=1\times 10^{-4}$, $weight\;decay=5\times 10^{-2}$, and batch size=4. We defined hyperparameters of the MLP and subblocks using grid search with the training set. This process was conducted with possible layer input, output channel sizes, model depth, and so on in an iterative manner to find the optimal hyperparameters.

### 2.5. Gradient-Weighted Class Activation Mapping

A visualization method known as gradient-weighted class activation mapping (Grad-CAM) was used to identify the factors affecting the prediction results of the proposed model [33]. This method used a gradient from a CNN-based model with a final convolutional layer containing high-level features and spatial information to highlight the important parts of an image for decision-making. This approach made it possible to describe the prediction process by determining how these features influence the model’s decision.

### 2.6. Statistical Analyses

Categorical variables were expressed as numbers and percentages, whereas continuous variables were summarized using means and standard deviations. We used either the χ^2^ test or Fisher’s exact test to compare categorical variables, whereas the Mann–Whitney U test was used for continuous variables. When comparing the baseline characteristics between the success and failure groups, statistical significance was set at *p* < 0.05. The optimal cutoff value for predicting weaning outcomes in the machine learning models was determined using Youden’s index.

Statistical analyses were conducted using Python 3.9, PyTorch 1.10.0, and an Nvidia RTX 3090 GPU. The backbone source code of the proposed model was obtained from a previous study [34].

## 3. Results

### 3.1. Baseline Characteristics

**Figure 3:**
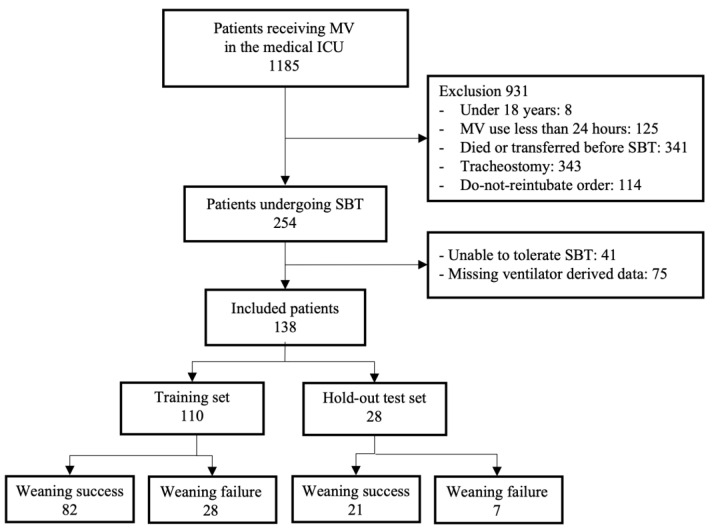
Participant enrollment flow diagram. MV, mechanical ventilation; ICU, intensive care unit; SBT, spontaneous breathing trial.

This study included 138 patients with an average age of 68.4 ± 15.1 years (Figure 3). Pneumonia was the most common reason for admission to the ICU, accounting for 71.7% of all cases. The weaning success and failure groups showed no significant differences in APACHE II scores and comorbidity, which are indicators of severity at the time of ICU admission. Similarly, no significant differences were observed in the duration of mechanical ventilation, number of previous weaning failures, and use of neuromuscular blocking agents, which are known risk factors for weaning failure. Arterial blood gas analysis was conducted to assess the patient’s oxygenation and ventilation just before the SBT, which showed that the PaCO_2_ level was slightly higher in the failure group. However, this difference was not statistically significant (Table 2).

### 3.2. Weaning Prediction Performance

**Figure 4:**
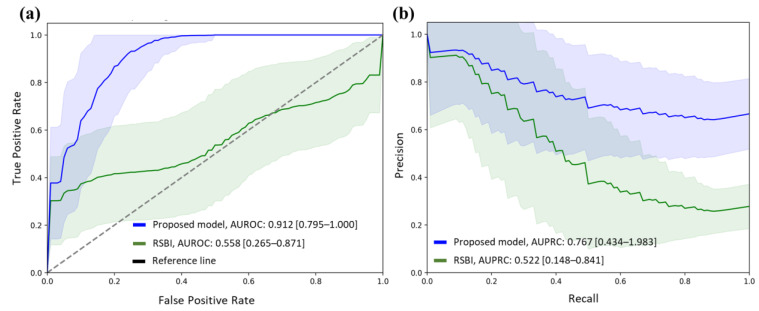
ROC and PRC with confidence intervals for ventilator weaning prediction model. (**a**) AUROC and (**b**) AUPRC performance of prediction model in the hold-out test set. ROC, receiver operating characteristic; PRC, precision-recall curve; AUROC, area under the ROC curve; AUPRC, area under the PRC; RSBI, rapid shallow breathing index.

The AUROC of the prediction model for weaning success was 0.912 (95% confidence interval [CI], 0.795–1.000) and the AUPRC was 0.767 (95% CI, 0.434–0.983) (Figure 4). The optimal cutoff value for predicting weaning outcomes, as confirmed using Youden’s index, was 0.475. Moreover, the proposed model had a parameter count of 17,124,721. The total computation time for the test set was measured to be 3.43613 s.

This study compares the traditional method (RSBI) of predicting successful weaning from mechanical ventilation with a machine learning model. The RSBI cutoff value of 105 breaths/min/L was used for comparison. The results demonstrated that the machine learning model outperformed the RSBI, with higher AUROC (0.912 vs. 0.558) and AUPRC (0.767 vs. 0.522) values (Table 3). Moreover, the machine learning model demonstrated superior discrimination ability compared with that of the RSBI in other predictive performance evaluation variables (Table 3).

### 3.3. Gradient-Weighted Class Activation Mapping

**Figure 5:**
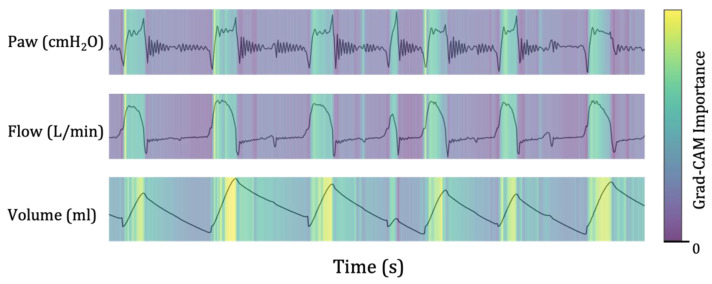
Gradient-weighted class activation mapping for ventilator-derived waveform data. Gradient-weighted class activation mapping (Grad-CAM) analysis can be used as a visualization tool to identify the areas of the waveform that are important for predicting weaning success. The Grad-CAM results are presented above the graph of each parameter. The brighter regions indicate areas that significantly influenced the model’s prediction results. Paw, airway pressure; Grad-CAM, the gradient-weighted class activation mapping.

Grad-CAM was used to determine which waveforms significantly impacted the prediction of weaning success or failure. The results of the Grad-CAM analysis using the ventilator-derived waveform data from a patient who was unable to wean are presented in Figure 5. The brightness intensity of the image corresponds to the degree of influence on the model’s prediction. Regions with higher brightness signify stronger influence on the model’s prediction. In Figure 5, the highlighted lesions were visible during the inspiratory phase, an active process involving the activation of neural pathways and contraction of inspiratory muscles. A rounded inspiratory flow and a significant decrease in airway pressure (Paw) during inspiration are signs of low ventilator assistance concerning the patient’s demands [35]. Discrepancies between the patient’s needs and the ventilator assistant are known to be associated with unfavorable outcomes [36]. Medical staff can interpret these signs as indicating that the patient is not yet ready for ventilator weaning and that the model’s weaning failure predictions are reliable.

**Figure 6:**
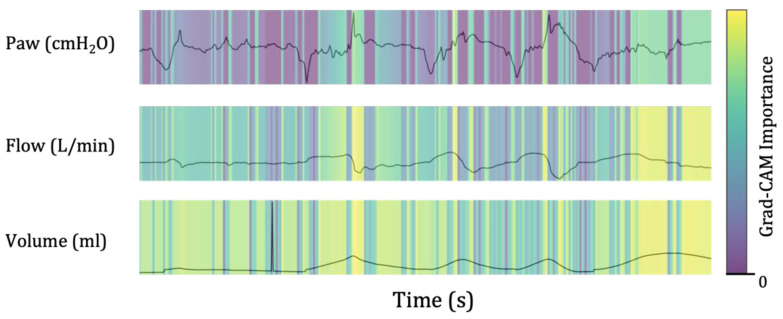
Example of misclassified errors using the predictive model. The patient was successfully weaned off the ventilator despite being predicted to fail. Figure 6 shows an artifact waveform caused by coughing, which was mistakenly identified as a sign of weaning failure by the machine learning model. The Grad-CAM tool allows medical staff to review the factors influencing the model’s decision and correct any errors. Paw, airway pressure; Grad-CAM, the gradient-weighted class activation mapping.

Grad-CAM is a helpful tool for medical professionals to identify errors in predictive judgment. For instance, if a patient is predicted to have failed weaning, but the medical staff confirms through Grad-CAM that the factor that affected the result was noise caused by coughing (Figure 6). Then, the prediction model’s results will not be trusted. Figure 6 showed a patient who succeeded in weaning, contrary to the prediction model results. Therefore, Grad-CAM can help medical professionals improve the accuracy of predictive models and provide better patient care.

## 4. Discussion

In this study, we developed a machine learning model to predict ventilator weaning outcomes in patients undergoing mechanical ventilation in a medical ICU. The machine learning model used only continuous ventilator parameters collected during SBT, which is routinely conducted during the weaning process. This study compared the predictive performance of the current model with that of the RSBI, a method already being used in clinical practice for predicting weaning. The predicted performance of the machine learning model was 0.912 for the AUROC and 0.767 for the AUPRC, showing superior results to those of the RSBI. In addition, Grad-CAM was used to visualize the waveform features that significantly influenced the prediction of weaning outcomes.

Many studies have explored the application of machine learning for predicting weaning outcomes (Table 4). With the advent of electronic health records, the extraction and integration of an array of patient information based on time series has become feasible, enabling studies that combine multiple modalities. A recent study developed a data-driven framework for predicting extubation outcomes in surgical ICU patients [18]. This framework included variable selection, prediction model, and Bayesian decision analysis processes. The model incorporated patient data, including demographic information, laboratory results, vital signs, and clinical scores such as the GCS, APACHE II, and RSBI. The authors also attempted to provide a comprehensive view of the extubation decision process, including respiratory, laboratory, biochemical, and neurological measurements. The developed model demonstrated a sensitivity of 0.830 and a specificity of 0.890 for prediction. Another single-center study introduced a machine learning tool for aiding in decision-making for extubation [37] that effectively integrates a variety of heterogeneous data, including patient demographics (age, sex, and body mass index), medical records (RSBI; respiratory rate oxygen index; GCS, Richmond Agitation-Sedation Scale, and APACHE II scores), medications (sedatives and analgesics), and respiratory event logs (ventilator mode, tidal volume, peak inspiratory pressure, plateau pressure, and FiO_2_). Although the model’s performance was internally validated, this prediction tool exhibited excellent predictive capabilities, with an AUROC of 98.3% and an accuracy of 94.6%. Other Medical Information Mart for Intensive Care (MIMIC)-III database studies predicted weaning outcomes with over 25 features, including demographics, comorbidities, vital signs, laboratory results, transfusions, fluids, medications, continuous renal replacement therapy, and the Charlson comorbidity index [20,21]. The results were promising with an AUROC of 0.80–0.94, indicating good predictive performance.

In our previous study [19], we used a conventional machine learning technique as the random forest classifier. For this classifier, we analyzed features from patients and extracted specific biosignal-based features from whole features. The Poincaré plot, sample entropy, and detrended fluctuation analysis were used in this process. Finally, the random forest classifier predicted weaning success using the selected features and RSBI. Using conventional machine learning techniques and feature analysis, we achieved high weaning prediction performance in our previous study, with an AUROC of 0.81. Despite the successful results, there were limitations. First, feature selection methods could have contributed to failure by not choosing important features or selecting the wrong features. Second, the weaning prediction performance was insufficient for practical use. In this study, the proposed method required neither feature selection of data nor cropping in the time domain. The proposed model used all features from the ventilator. Thus, our method can reduce the prediction error produced by using feature selection.

Many studies have shown that utilizing multiple variables from diverse modalities reduces errors stemming from using only a single-modality approach and improves prediction performance through the incorporation of various types of information. Therefore, machine-learning models that predict weaning outcomes have been developed using a wide range of input features. The use of various variables can enhance the performance of a predictive model. However, this may hinder the real-time application of the model in clinical practice. Continuously monitoring variables, such as clinical scores (e.g., APACHE II score and Charlson comorbidity index) and cumulative doses of drugs is particularly challenging for medical staff, especially when a patient’s condition is rapidly fluctuating.

In our study we aimed to develop a predictive model by devising a method that could serve as an intuitive aid for medical staff when making weaning decisions at the bedside for patients in ICU. The breathing pattern observed in routinely conducted SBTs during the weaning process is an excellent indicator of weaning outcomes [38,39,40]. Patients who are unable to successfully wean from mechanical ventilation often exhibit irregular breathing patterns during SBTs. This can be attributed to factors, such as inadequate volume capacity, unstable hemodynamic status, and respiratory muscle weakness [7,41]. These unstable respiratory mechanisms can lead to an oxygenation-ventilation imbalance, ultimately resulting in weaning failure [42,43]. Continuous ventilator monitoring data provide a more comprehensive view of a patient’s breathing patterns throughout the entire course of the SBT instead of relying on a single-point approach, such as those adopted by classic weaning indices. Therefore, by lowering ventilator support during the SBT and observing the dynamic changes in the patient’s breathing pattern, weaning outcomes can be predicted in real-time at the bedside.

Although artificial intelligence has the advantage of being able to handle vast data from various modalities, it still requires improvement to compensate for the unpredictable situations and countless errors that occur in actual clinical settings. High-quality and reliable input data are essential for developing a more accurate model and improving its performance. Although integrating and analyzing multiple modalities may enhance the performance of predictive models, input data characteristics must be generalized to collect and use data from various medical institutions, and a high-quality protocol must be maintained. Although our study used only a single measurement (ventilator data), our predictive model achieved a good prediction performance by ensuring reliable, high-quality data. In the ICU, many events affect a patient’s clinical data, such as position changes and suctioning. Hence, efforts were made to avoid suction as much as possible during the SBT to increase the accuracy of the collected ventilator data and mitigate potential noise that could occur during the SBT. We collected data approximately 30 min into the SBT owing to the relatively short time requirement to allow for quality control procedures. The strength of our model lies in its ability to produce significant effects using a relatively small amount of data.

Another strength of this study is that the time-domain sequential ventilator data were transformed into two-dimensional image data using a CNN-based learning network. Continuous ventilator waveform data were used as graphical information. The decision-making process was visually explained through a localization map highlighting the essential decision-making areas using the Grad-CAM technology. As errors in judgment for treating severely ill patients could affect the patient’s prognosis, using a clinical decision support system based on machine learning in clinical settings could pose certain limitations. However, the model developed in this study could reduce predictive judgment errors and facilitate its implementation in clinical practice by presenting the waveform features that form the basis of the decision to medical staff. In other words, the model’s explainability helps clinicians make better decisions, thereby improving patient outcomes. As a result, medical staff utilize Grad-CAM to analyze the part of the data that could affect the model’s prediction results and determine whether the features are meaningful (Figure 5 and Figure 6).

Despite the impressive performance of the proposed model, it had certain limitations that warrant consideration. First, the limited dataset size raises the possibility of overfitting. External validation is necessary to ensure accurate evaluation. Second, the number of patients experiencing weaning failure in the dataset was insignificant, and thus, it might have hindered the model’s ability to perfectly learn failure patterns. Another limitation in machine learning models is that those other than CNN may not be applicable due to the varying length of input data. CNN is more suitable for handling diverse data than Recurrent neural network (RNN)-based models or transformers, which can only handle fixed data lengths. Moreover, RNNs and transformers demand substantial computational resources, and notably, transformers require much larger datasets than other deep learning networks. Further studies are necessary to explore the potential of different models in the future. Finally, we could visualize the features that influenced the decision-making process of the predictive model. It is essential to remember that it can be challenging to confirm a direct connection between these features and the outcomes. In future studies, we must still pinpoint a distinct ventilator waveform pattern that can differentiate between successful and unsuccessful weaning results.

## 5. Conclusions

We developed a model to predict weaning outcomes using only continuous monitoring parameters acquired from the ventilator during SBT. This model demonstrated excellent predictive performance (AUROC, 0.912; AUPRC, 0.767). Furthermore, its ability to visualize the features that affect the outcome and present them to the medical staff holds great potential for reducing potential errors that may arise when applying machine learning models in a clinical setting. Therefore, it is expected to be a promising tool for medical staff treating ventilator patients, thereby significantly reducing their burden by supporting real-time decision-making for weaning and improving patient prognosis. In future, we need to identify a specific ventilator waveform pattern distinguishing between successful and failed weaning outcomes.

## Figures and Tables

**Table 1:** Specification of the feature extractor and classifier.

	MobileNetV3 Operator	Expand Size	Out Channel	Activation Function
Feature extractor	Conv2d	-	16	H-Swish
	bneck, 3 × 3	16	16	ReLU
	bneck, 3 × 3	64	24	ReLU
	bneck, 3 × 3	72	24	ReLU
	bneck, 5 × 5	72	32	ReLU
	bneck, 5 × 5	96	32	ReLU
	bneck, 5 × 5	96	32	ReLU
	bneck, 3 × 3	192	64	H-Swish
	bneck, 3 × 3	160	64	H-Swish
	bneck, 3 × 3	144	64	H-Swish
	bneck, 3 × 3	144	64	H-Swish
	bneck, 3 × 3	384	88	H-Swish
	bneck, 3 × 3	528	88	H-Swish
	bneck, 5 × 5	528	120	H-Swish
	bneck, 5 × 5	720	120	H-Swish
	bneck, 5 × 5	720	120	H-Swish
	Conv2d, 1 × 1	-	720	H-Swish
	Pool, 7 × 7	-	-	-
Classifier	Conv2d, 1 × 1, NBN	-	1280	H-Swish
	Conv2d, 1 × 1, NBN	-	1	-

Conv2d, two-dimensional convolution layer; H-Swish, hard swish; bneck, bottleneck; ReLU, rectified linear unit; NBN, non-bottleneck.

**Table 2:** Baseline characteristics of study participants according to weaning outcomes

	Total (N = 138)	Success Group (N = 103)	Failure Group (N = 35)	*p* Value
Age, mean ± SD, y	68.4 ± 15.1	68.9 ± 14.6	67.0 ± 16.6	0.507
Sex, male/female, n	87/51	67/36	20/15	0.403
Body weight, mean ± SD, kg	59.7 ± 14.3	60.8 ± 15.0	56.6 ± 11.5	0.136
Height, mean ± SD, cm	164.2 ± 9.6	164.1 ± 9.9	164.4 ± 8.6	0.899
BMI, mean ± SD, kg/m^2^	22.1 ± 4.6	22.5 ± 4.8	20.9 ± 3.7	0.072
Main cause of ICU admission, n, %				0.869
Pneumonia	99 (71.7)	76 (73.8)	23 (65.7)	
COPD/Asthma AE	10 (7.2)	7 (6.8)	3 (8.6)	
Pulmonary hemorrhage	3 (2.2)	2 (1.9)	1 (2.9)	
Sepsis	4 (2.9)	3 (2.9)	1 (2.9)	
Gastrointestinal bleeding	2 (1.4)	2 (1.9)	0 (0)	
Neurologic disease	2 (1.4)	1 (1.0)	1 (2.9)	
Pulmonary edema	9 (6.5)	6 (5.8)	3 (8.6)	
Others	9 (6.5)	6 (5.8)	3 (8.6)	
APACHE II score, mean ± SD	22.6 ± 8.3	23.0 ± 8.4	21.5 ± 7.9	0.332
Comorbidity, n, %				
HTN	62 (44.9)	47 (45.6)	20 (42.9)	0.776
Diabetes mellitus	41 (29.7)	35 (34.0)	6 (17.1)	0.060
COPD	13 (9.4)	8 (7.8)	5 (14.3)	0.315
Chronic lung disease	40 (29.0)	26 (25.2)	14 (40.0)	0.096
Neurological disease	46 (33.3)	34 (33.0)	12 (34.3)	0.890
Cancer	27 (19.6)	22 (21.4)	5 (14.3)	0.362
Renal disease	15 (10.9)	13 (12.6)	2 (5.7)	0.355
Liver disease	12 (8.7)	10 (9.7)	2 (5.7)	0.730
Residence type before admission				0.411
Home	99 (71.7)	72 (69.9)	27 (77.1)	
Hospital or nursing home	39 (28.3)	31 (30.1)	8 (22.9)	
ABGA before SBT				
PaO_2_	106.6 ± 32.1	107.8 ± 29.7	103.3 ± 38.5	0.478
PaCO_2_	37.6 ± 10.2	36.6 ± 9.9	40.3 ± 10.8	0.063
PF ratio	319.5 ± 100.1	325.4 ± 92.8	302.1 ± 118.6	0.235
Length of mechanical ventilation before SBT, mean ± SD	7.7 ± 6.2	7.53 ± 6.5	8.2 ± 4.8	0.598
Prior failed weaning attempt	20 (14.5)	13 (12.6)	7 (20.0)	0.284
Use of NMBAs	25 (18.1)	18 (17.5)	7 (20.0)	0.738

Values are presented as the mean with standard deviation (SD) or number (%). BMI, body mass index; ICU, intensive care unit; COPD, chronic obstructive pulmonary disease; AE, acute exacerbation; APACHE II, Acute Physiologic and Chronic Health Evaluation II; HTN, hypertension; ABGA, arterial blood gas analysis; SBT, spontaneous breathing test; MV, mechanical ventilation; PaO_2_, partial pressure of oxygen in the arterial blood; PaCO_2_, partial pressure of carbon dioxide; PF ratio, ratio of arterial oxygen partial pressure to fraction of inspired oxygen; NMBAs, neuromuscular blocking agents.

**Table 3:** Comparison of performance in the hold-out test set.

	AUROC	AUPRC	Sensitivity	Specificity	NPV	PPV	Accuracy	F1 Score
ML model	0.912 (0.795–1.000)	0.767 (0.434–0.983)	0.857 (0.555–1.000)	0.808 (0.619–0.952)	0.943 (0.800–1.000)	0.608 (0.286–0.889)	0.821 (0.679–0.929)	0.698 (0.400–0.909)
RBSI	0.558 (0.265–0.871)	0.522 (0.148–0.841)	0.423 (0.000–0.833)	0.907 (0.762–1.000)	0.820 (0.667–0.958)	0.607 (0.001–0.999)	0.783 (0.607–0.929)	0.476 (0.001–0.824)

AUROC, area under the receiver operating characteristic; AUPRC, area under the precision-recall curve; NPV, negative predictive value; PPV, positive predictive value; ML, machine-learning; RSBI, rapid shallow breathing index.

**Table 4:** Summary of studies using machine learning models to predict mechanical ventilator weaning.

Authors	Data Source	Variables	ML Model	Performance	Study Design
Tsai et al. [18]	Surgical ICU (n = 704)	17 features Demographics (e.g., sex and weight) Laboratory results (e.g., WBC, platelets, glucose, sodium) Clinical scores (GCS and APACHE II scores, RSBI) Ventilator parameter (maximal inspiratory pressure) Vital signs (HR, blood pressure)	Machine learning ensemble	Sensitivity: 0.830, Specificity: 0.890	Retrospective
Fabregat et al. [37]	ICU (n = 697)	20 features Demographics (age, sex, and body mass index) Medical records (e.g., RSBI; ROX index; GCS, RASS, and APACHE II scores) Medications (cumulative/administered dose of sedatives and analgesics) Ventilator parameter (e.g., peak inspiratory pressure, plateau pressure, and FiO_2_).	Support vector machine	AUC: 0.98	Retrospective
Zhao et al. [20]	MIMIC-IV (n = 16,189)	19 features Demographics (age and body mass index) Medical records (stroke and urine output) Vital signs (e.g., HR, RR, saturation, temperature, and CVP) Respiratory parameters (e.g., tidal volume, PEEP, mean airway pressure, and PSV level) Laboratory results (pH) Medications (e.g., antibiotics)	Categorical boosting (CatBoost)	AUC: (internal) 0.835, (external) 0.803	Retrospective (development), Prospective (validation)
Jia et al. [21]	MIMIC-III (n = 2299)	25 features Demographic information (e.g., age, admission type, reason for intubation, RASS score) Vital signs (e.g., HR, RR) Laboratory results (e.g., sodium level, serum anion gap, partial pressure of oxygen/carbon dioxide) Respiratory parameters (e.g., duration of mechanical ventilation, tidal volume)	1d-CNN	AUC: 0.94	Retrospective
Park et al. [19]	Medical ICU (n = 89)	10 features Vital signs (ECG, respiratory impedance, PPG, arterial blood pressure, HR, and RR) Ventilator parameters (tidal volume, inspiratory time, IE ratio)	Random forest	AUC: 0.81	Retrospective
Park et al.	Medical ICU (n = 138)	28 features Ventilator parameters (waveform: pressure, flow, and volume. numerical: inspiratory tidal volume, spontaneous expiratory tidal volume, breathing frequency, IE ratio, airway occlusion pressure)	CNN	AUC: 0.912	Retrospective

1d-CNN, one-dimensional convolutional neural network; APACHE, Acute Physiologic and Chronic Health Evaluation; AUC, area under the receiver operating characteristic curve; CNN, convolutional neural network; CVP, central venous pressure; ECG, electrocardiogram; FiO_2,_ fraction of inspired oxygen; GCS, Glasgow Coma Scale; HR, heart rate; ICU, intensive care unit; IE ratio, the ratio of inspiratory and expiratory time; MIMIC-IV, Medical Information Mart for Intensive Care-IV; ML model, machine learning model; PEEP, positive end-expiratory pressure; PPG, photoplethysmogram; PSV, pressure support ventilation; RASS, Richmond Agitation Sedation Scale; ROX index, respiratory rate oxygen index; RR, respiratory rate; RSBI, rapid shallow breathing index; WBC, white blood cell.

## Data Availability

The data presented in this study are available on request from the corresponding author.

## Supplementary Materials

The following supporting information can be downloaded at: https://www.mdpi.com/article/10.3390/bioengineering10101163/s1, Table S1: Variables used to develop predictive models for ventilator weaning outcome.
