# Supplementary material for: Development of a Machine Learning Model for Predicting Weaning Outcomes Based Solely on Continuous Ventilator Parameters during Spontaneous Breathing Trials
Source: Bioengineering (Basel). 2023 Oct 5;10(10):1163. doi: 10.3390/bioengineering10101163 (PMC10604888; doi:10.3390/bioengineering10101163)
Supplement: Supplementary file 1 [file bioengineering-10-01163-s001.zip › bioengineering-2627336-supplementary.pdf]

Supplementary Table S1. Variables used to develop predictive models for ventilator weaning outcome

Parameter	Definition
**Numeric**	
VTI (ml)	Inspiratory tidal volume
VTE (ml)	Expiratory tidal volume
ExpMinVol (l/min)	Expiratory minute volume
Insp flow (l/min)	Peak inspiratory flow
VTE spont (ml)	Spontaneous expiratory tidal volume
Vt leak (ml)	Leakage volume
Exp flow (l/min)	Peak expiratory flow
P peak (cmH_2O)	Peak airway pressure
P mean (cmH_2O)	Mean airway pressure (Ppeak – PEEP/CPAP)
PEEP/CPAP (cmH_2O)	Positive end expiratory pressure
P0.1 (cmH_2O)	Airway occlusion pressure
Variability Index (%)	Variability of volume and timing, the coefficient of variation (standard deviation/mean) of the tidal volume/inspiratory time index
f total (b/min)	Total breathing frequency
f spont (b/min)	Spontaneous breath frequency
TI (s)	Inspiratory time
TE (s)	Expiratory time
I:E	Ratio of inspiratory time to expiratory time
Oxygen (%)	Oxygen concentration to be delivered
Rinsp (cmH_2O/l/s)	Resistance to inspiratory flow caused by the endotracheal tube and the patient's airways, during inspiration
Rexp (cmH_2O/l/s)	Resistance to expiratory flow caused by the endotracheal tube and major airways during exhalation
Cstat (ml/cmH_2O)	Static compliance
Rcexp (s)	Expiratory time constant
Rcinsp (s)	Inspiratory time constant.
PTP (cmH_2O*s)	Inspiratory pressure time product
WOB	Work of breathing
**Waveform**	
Paw (cmH_2O)	Pressure waves versus time
Flow (L/min)	Flow waves versus time
Volume (ml)	Volume waves versus time
